# Lipedema: A Disease Triggered by M2 Polarized Macrophages?

**DOI:** 10.3390/biomedicines13030561

**Published:** 2025-02-23

**Authors:** Thomas Grewal, Sally Kempa, Christa Buechler

**Affiliations:** 1 School of Pharmacy, Faculty of Medicine and Health, University of Sydney, Sydney, NSW 2006, Australia; thomas.grewal@sydney.edu.au; 2 Department of Plastic, Hand, and Reconstructive Surgery, University Hospital Regensburg, 93053 Regensburg, Germany; sally.kempa@klinik.uni-regensburg.de; 3 Department of Internal Medicine I, University Hospital Regensburg, 93053 Regensburg, Germany

**Keywords:** lipedema, macrophage, adipocyte

## Abstract

**Background/Objectives**: Lipedema is a progressive disease that results in the bilateral and symmetrical accumulation of subcutaneous fat in the legs and/or arms, affecting almost exclusively women. **Methods**: A comprehensive review of the peer-reviewed literature was conducted between November 2024 and February 2025. **Results**: The pathophysiology of lipedema is complex and, especially in the early stages, shows similarities to obesity, involving adipocytes, adipose tissue-resident macrophages, and endothelial cells. In lipedema, systemic levels and the adipocyte expression of the classical adipokines adiponectin and leptin appear normal, while it remains unclear if markers of inflammation and oxidative stress are increased. Macrophages in the adipose tissue of patients have an anti-inflammatory M2 phenotype and express high levels of the scavenger receptor CD163. These cells affect adipogenesis and seem to have a central role in adipose tissue accumulation. Increased lymphatic and blood vessel permeability are comorbidities of lipedema that occur in early disease states and may contribute to disease progression. **Conclusions**: This review summarizes our current understanding of the pathophysiology of lipedema with a focus on the role of stromal vascular localized M2 macrophages.

## 1. Introduction

Lipedema is a relatively common condition affecting almost exclusively women with a 10% prevalence. The pathophysiology of lipedema is largely unknown and is influenced by genetic factors with a positive family history in >80% of patients [1]. The condition typically develops during puberty, pregnancy, or menopause, suggesting that hormones such as estrogen play a role in disease manifestation [2,3]. Indeed, one survey found 48% and 41% of women experiencing lipedema symptoms during puberty and pregnancy, respectively [4].

As standard blood or imaging tests are lacking, lipedema is rather diagnosed upon a comprehensive review of the patient’s medical history and a physical examination [5]. Affected subjects are often obese with a body mass index (BMI) ≥ 30 kg/m^2^ yet lack metabolic abnormalities commonly associated with obesity. Hence, lipedema patients remain insulin-sensitive, display normal blood pressure and almost normal blood lipid levels, and do not store excess fat in the liver [6,7,8]. Furthermore, these individuals exhibit normal adipokine levels, such as adiponectin and leptin, which are typically reduced (adiponectin) or elevated (leptin) in obese patients and produced almost exclusively by adipocytes. Likewise, C-reactive protein (CRP) levels, a clinical marker of inflammation and commonly elevated in obesity, appear normal [6].

Lipedema could be classified as a metabolically healthy type of obesity in which excess fat is stored predominantly in the subcutaneous adipose tissue of the legs, a process considered an effort to protect organs such as muscle and liver from steatosis [9,10]. Importantly, metabolically healthy but obese individuals display less visceral fat compared to BMI-matched obesity patients [6], the latter representing a known risk factor for metabolic diseases such as type 2 diabetes and fatty liver disease [9,10]. However, in contrast to lipedema, metabolically healthy obesity occurs in both women and men, with a slightly higher prevalence in women, which is plausible because more women are obese [7]. In US adults, about 14% of men and 16% of women were defined as metabolically healthy obese [7], a finding recently supported in a study of another population group [11]. Thus, given that lipedema almost exclusively affects women, causing pain and generally resisting dietary and exercise measures [3,5,12], it may not be considered metabolic healthy obesity.

Lipedema is a long-lasting and progressive condition that affects adipose tissue, resulting in a bilateral and symmetrical but disproportionate accumulation of subcutaneous fat in legs and/or arms, excluding hands and feet. Patients experience tension, and haptic perception of the affected limbs is painful [5]. Although the pathophysiological basis for the different morphologies of lipedema is currently unknown, five types of lipedema can be distinguished according to the distribution of the excess adipose tissue [3]. Three clinical stages of lipedema have been defined, and patients with stage 3 and lipolymphedema are classified by some authors as stage 4 lipedema [1,3,5]. The final stage of this disease is associated with a higher prevalence of obesity, thicker skin, and more adipose tissue [1]. Patients with lipedema develop bruising spontaneously and after minimal trauma [1]. Common comorbidities in patients with lipedema are depression, allergies, and venous disease. Polycystic ovary syndrome and hypothyroidism also often occur [1,13]. A study of >700 women with lipedema reported a higher incidence of flu-like symptoms, joint hypermobility, cool skin, varicose veins, and fatigue compared to age- and BMI-matched controls [4].

Dyslipidemia has also been reported in patients with lipedema [1]. Plasma total cholesterol and low-density lipoprotein cholesterol levels were elevated in women with lipedema [14,15]. Plasma triglyceride amounts were also higher, whereas high-density lipoprotein levels were in the normal range [15]. The prevalence of dyslipidemia in stage 1, 2, and 3 patients was similar [1], indicating that pathological lipid profiles were not related to obesity and disease progression. However, a lipidomic comparison of the serum of a limited number (n = 8) of age- and BMI-matched lipedema patients and controls did not reveal any differences in the serum lipidome [16]. In contrast to the findings described above, lipoprotein levels and the lipid composition of the lipoprotein particles of patients and the controls were comparable [17]. On the other hand, implicating changes in serum lipids possibly related to hepatic complications and also observed in patients with liver disease, aminotransferase levels were higher in women (n = 26) with lipedema compared to age- and BMI-matched controls (n = 13) [14,18]. However, additional evidence for liver dysfunction in lipedema patients is lacking, as gamma-glutamyl transferase and lipase levels were similar between these groups [14]. Furthermore, in a relatively large cohort of Italian lipedema patients, liver function parameters were in the normal range [1].

Interestingly, people with lipedema are less likely to develop hyperglycemia. In female lipedema patients, hemoglobin A1c amounts were lower, and fasting insulin levels were higher compared to their age- and BMI-matched controls [14]. In support of this observation, patients with lipedema had a lower incidence of hyperglycemia, metabolic syndrome, and type 2 diabetes compared to normal female controls [1].

Current treatment options focus on symptom relief and are based on comprehensive decongestive therapy. Conservative measures can reduce the discomfort associated with tension and pressure and minimize the likelihood of hematoma formation [5]. The efficacy of weight loss and increased physical activity in improving symptoms and pain in patients with lipedema is still in question [12]. In one study, more than 50% of patients reported no improvement in symptoms with exercise and/or diet [4]. However, a meta-analysis evaluating the impact of a prolonged ketogenic diet for ~16 weeks revealed weight loss and reduced pain sensitivity [19]. Weight loss achieved by a low-carbohydrate diet was also shown to improve pain sensitivity in a cohort of 70 female patients [20]. Further studies are needed to investigate whether specific diets improve lipedema symptoms.

If conservative therapies fail to improve symptoms, liposuction has also been considered [3,5,13]. A recent meta-analysis of seven trials showed significant improvements in spontaneous pain, swelling, bruising, and mobility in patients with lipedema who underwent liposuction. However, more than 50% of these patients continued to require conservative treatment after surgery [21]. Nevertheless, improvements in spontaneous pain, edema, tenderness, bruising, mobility, and overall dysfunction were maintained at 4, 8, and 12 years follow-ups compared to the pre-operative baseline [22].

In the following, we will summarize our current knowledge regarding the pathophysiology of lipedema, focusing on adipogenesis and the various cell types influencing this process, stromal vascular cells, endothelial cells, and adipose tissue resident macrophages.

## 2. Adipokines and Other Systemic Metabolites

### 2.1. Adipokines

As outlined above, lipedema is characterized by the accumulation of adipose tissue, which prompted investigations to determine if systemic adipokines could be used as biomarkers for the diagnosis of this disease. Based on the common traits observed in obese patients, research up to date has focused on the analysis of adiponectin, leptin, visfatin, and resistin as potential biomarkers for lipedema. Adiponectin is produced almost exclusively by adipocytes and has many beneficial effects on metabolism in distal tissues [23,24]. In addition, adiponectin acts as an autocrine factor in adipose tissue and facilitates the proliferation and differentiation of preadipocytes into adipocytes, increases the expression of genes associated with adipogenesis, and improves both the lipid accumulation and insulin sensitivity of adipocytes [24]. In obesity, serum adiponectin levels are reduced, contributing to insulin resistance and hepatic steatosis [23].

Leptin is a satiety hormone that has inflammatory and fibrotic effects. In obese patients, leptin levels are commonly increased, and leptin resistance may contribute to hyperleptinemia and impaired appetite control [25]. Visfatin, also known as pre-B-cell colony-enhancing factor, is responsible for the production of nicotinamide mononucleotide, which is a precursor for the biosynthesis of nicotinamide adenine dinucleotide [26]. Secreted visfatin has inflammatory and profibrotic properties, and in obese individuals, visfatin serum levels and adipocyte expression are significantly increased [27,28]. Resistin is secreted by adipocytes and immune cells, and its systemic levels increase in the obese and exert pro-inflammatory activities in humans, contributing to insulin resistance [29].

However, despite the accumulation of subcutaneous fat, the limited information that is currently available documents that women with lipedema have rather normal levels of adiponectin, leptin, visfatin, and resistin (Table 1).

### 2.2. Other Systemic Proteins and Metabolites

Biomarkers for lipedema diagnosis are still lacking, and cytokine, CRP, and amino acid levels in the serum of patients and controls have been considered for this purpose. In most patients with stage 3 disease, higher circulating levels of the inflammatory mediators tumor necrosis factor ligand superfamily member 14, caspase 8, S100-A12, eukaryotic translation initiation factor 4E binding protein 1, adenosine deaminase, and chemokine ligand 2 and the oxidative stress markers superoxide dismutase, malondialdehyde, and catalase were detected [14]. Plasma interleukin-6 (IL-6) and CRP levels of these patients were normal [14,31]. Another study found higher serum levels of IL-1, IL-28, and IL-29 in patients with lipedema [16]. One year after liposuction, the volumetric changes in the legs of the five women included in this study did not significantly decline post-operatively. The amounts of interferon alpha, a protein which inhibits adipogenesis, and IL-34, known to contribute to metabolic abnormalities, were decreased in the serum of these patients [16]. IL-18 and lipocalin-2 levels were normal in lipedema patients [15]. There was also evidence that lipedema in advanced disease states was associated with increased amounts of systemic CRP [1].

Beyond the determination of adipo-/cytokine levels and several parameters relevant for glucose and lipid homeostasis, serum metabolomic profiling of 25 women diagnosed with lipedema and 25 obese women showed decreased levels of histidine and phenylalanine and increased levels of pyruvic acid in lipedema patients. Receiver operating characteristic curves demonstrated excellent diagnostic accuracy for histidine, phenylalanine, and pyruvate concentrations in discriminating between individuals with lipedema and those with obesity without lipedema [17]. Histidine and phenylalanine are essential amino acids involved in numerous metabolic processes [32,33], and their reduced serum concentrations probably reflect changes in protein metabolism, amino acid usage, and associated pathways. Elevated levels of pyruvate in lipedema may reflect reduced metabolism through the citric acid cycle [34], which can lead to a variety of metabolic abnormalities. In adipocytes, perturbing the flux of pyruvate significantly reduced triglyceride accumulation and altered glycolysis, lipolysis, and de novo fatty acid synthesis [35].

## 3. Adipogenesis, Adipocyte Size, and Fibrosis

### 3.1. Adipogenesis

The expression of adiponectin and leptin are induced during adipogenesis and have therefore served as adipocyte differentiation markers [36]. Thus, the adipokine levels of adipose-derived stem cells from subcutaneous thigh and abdominal fat of age- and BMI-matched controls and lipedema patients were compared. In these studies, adipocytes derived from femoral stem cells of lipedema patients (15% stage 1, 70% stage 2) displayed higher leptin expression compared to the controls [37]. In line with these findings, the levels of *peroxisome proliferator-activated receptor gamma* (PPAR-γ), an essential transcription factor for adipogenesis, were higher in adipocytes derived from the abdominal fat of patients [37]. On the other hand, when analyzing the subcutaneous fat of the thigh, the expression of adipogenic markers decreased with higher disease stages [38].

The difficulty to develop a common theme for adipogenesis in lipedema is further exemplified in studies that utilized lipedema adipose-derived stem cells from patients with stage 2 disease, which showed impaired adipogenesis, a marked decrease in cytoplasmic lipid accumulation, and significantly reduced levels of adiponectin and leptin in adipocyte supernatants compared to the controls [39]. Adipogenic differentiation of isolated stromal vascular cells in stage 2–3 lipedema (n = 30: 55% stage 2, 24% stage 2–3, 21% stage 3; 22 controls) was also impaired [40]. Notably, the chondrogenic and osteogenic differentiation of these cells was normal [40].

While some of the abovementioned and cell-based studies implicate impaired adipogenesis in lipedema, 3D spheroids derived from adipose-derived stem cells from patients (n = 8; >85% stage 2–3) exhibited expression levels of adipogenic genes, in particular adiponectin, lipoprotein lipase, PPAR-γ, and glucose transporter type 4 that were similar to spheroids from healthy controls (n = 8; with lower BMI: 27.3 ± 1.0 vs. 30.4 ± 1.2 kg/m^2^ in controls) [41]. In line with these observations, the adipogenic capacity of adipose tissue-derived stem cells from non-obese and obese donors with or without lipedema did not correlate with donor body mass index and showed no significant differences between groups [42].

However, some yet to be identified differences in their potency to differentiate along the adipogenic pathway appear to exist, as in vitro differentiated adipocytes from non-obese lipedema donors showed a significant increase in the expression of *PPAR-γ*, CD36, and *fatty acid binding protein 4* compared to non-obese controls. The ratio of adiponectin and leptin levels was significantly lower in adipocytes from obese lipedema donors compared to their non-obese counterparts [42]. Consistent with this observation, in vitro differentiation of adipocyte-derived stem cells from lipedema patients (14 patients with stage 2/3, BMI 27 ± 10 kg/m^2^; 10 controls, BMI 26 ± 11 kg/m^2^) indicated a higher capacity for adipogenesis and increased fat storage [43].

The underlying causes for the discordant results described above remain to be clarified. With the exception of the study conducted by Ishaq et al. [43], who excised adipose tissue from the lower limbs, lipoaspirates were used to obtain adipose tissue cells [39,40,42]. Furthermore, different set-ups and experimental conditions, such as variations in media and growth conditions for adipogenic differentiation, may have contributed to inconsistent findings [39,40,42,43]. Ernst et al. postulated that after 7 and 14 days of in vitro differentiation, lipedema preadipocytes displayed a reduced capacity to accumulate lipids compared to non-lipedema controls, yet these differences disappeared after prolonged adipocyte differentiation periods (21 days) [42]. In contrast, Ishaq and coworkers reported improved adipogenesis in lipedema, differentiating cells for 14 days [43], while Priglinger et al. reported impaired adipogenesis, differentiating cells for 21 days [40]. Several other factors may have contributed to different outcomes in these studies. This includes the cell density during adipogenic differentiation, which can profoundly alter the production of endocrine factors [44]. The use of different differentiation markers to characterize mature adipocytes may also add to interpretations that cannot be compared in a straightforward manner. In particular, lipid accumulation is not a defining feature of mature adipocytes, and lipid storage may be uncoupled from adipogenesis [45]. Therefore, for better comparison, future in vitro studies should utilize a marker panel describing the characteristics of mature adipocytes. Notably, most of the abovementioned studies were based on small cohorts, and random variations when having a small number of samples can lead to conflicting results [46]. Furthermore, defining parameters for suitable controls in lipedema studies is complex, and controls are generally matched for BMI and age and should be otherwise healthy. However, recruitment of women to take part as controls in these studies remains a challenge.

Hence, a clear-cut view of potential changes related to adipocyte differentiation in lipedema is still lacking. Limitations of the studies listed above include differences in the experimental procedures to culture and differentiate stem cells and not taking into account the effects of the stromal vascular cell fraction (SVF), which, when isolated from lipedema patients, promoted adipocyte differentiation and lipid accumulation of adipose tissue-derived stem cells [47]. Hence, in summary, it is currently unclear whether adipogenesis is impaired in lipedema, and the potentially critical contribution of disease-related differences in the composition of the microenvironment that surrounds adipocytes (e.g., SVF) in this process still remains understudied.

### 3.2. Adipocyte Volume and Fibrosis

In adults, the generation of new adipocytes is part of the normal turnover of adipose tissue. The size of adipocytes increases until a state of moderate obesity is reached. Beyond this threshold, further increases in fat mass are primarily associated with an increase in the number of adipocytes [48]. This process, fat expansion achieved by hyperplasia, is considered a more favorable mechanism than adipocyte hypertrophy, which is associated with hypoxia, immune cell infiltration, and possibly adipose tissue fibrosis [9].

Adipose tissue expansion is achieved by hyperplasia and hypertrophy, the latter being dependent on adipocyte inflammation [49,50]. However, it remains unclear which of these pathways is predominant in lipedema. Several studies have addressed potential pathways that may be responsible for increased fat mass in lipedema, yet as outlined below, generalizing conclusions are still lacking. For instance, adipose-derived stem cells from patients with stage 2-3 disease were found to proliferate better than cells from controls, and this may contribute to adipose tissue growth [39,43]. However, another study showed comparable proliferation behavior of adipose-derived stem cells from patients and controls [37]. Furthermore, while one study found similar sizes of adipocytes in early stage lipedema and controls [38,51], others found lipedema adipocytes to be hypertrophic [49] (Table 2). Hence, similarities to hypertrophic mechanisms relevant in obesity may exist, and comparable adipocyte areas in obese lipidemic patients (mostly stage 2) and obese controls have been reported [49]. Other studies with mostly stage 2 and 3 patients have also observed adipocyte hypertrophy [15,16,52], indicating that in advanced lipedema, which is often associated with a higher BMI, lipidemic fat appears to grow by hypertrophy (Table 2).

A meta-analysis of the studies listed in Table 2 showed that most reports described adipocyte hypertrophy in fat tissue of women with lipedema. Greater fat mass is known to be associated with larger adipocytes [53]. Hence, future analysis that will assess adipocyte size relative to fat mass may show whether larger cell size correlates with adipose tissue mass of patients and controls.

Adipose tissue fibrosis hinders the growth of adipose tissue and leads to the deposition of fat in peripheral tissues, which subsequently causes insulin resistance [9]. However, it is still an unresolved issue whether fibrosis develops in lipedemic fat tissues. Along these lines, the production of extracellular matrix proteins that are induced in fibrotic tissues, such as collagen and fibronectin, was comparable in lipedema and control spheroids [41]. In contrast, a transcriptomic analysis of subcutaneous fat tissue revealed an upregulation of *lumican* [54], which is a component of the extracellular matrix and is increased in fibrosis [55]. Others also reported higher levels of intercellular fibrosis [15] and interstitial collagen [38] in lipedema, which could indeed indicate that adipose tissue fibrosis develops in the fat tissue of lipedema. Fibrotic adipose tissue is resistant to weight loss [56], and patients with lipedema hardly lose fat mass upon calorie-restricted diets [4]. However, further studies are needed to better comprehend the physiological requirements for adipocyte fibrosis to occur and unravel how the extent and composition of the extracellular matrix may contribute to pathophysiological effects in lipedema.

## 4. Stromal Vascular Cells and M2 Macrophages

Macrophages play a pivotal role in the proper functioning of the immune system and are distributed throughout the body, including adipose tissue. Most strikingly, obesity is associated with an increased infiltration of macrophages in fat tissue. This is accompanied by a phenotypic switch in adipose tissue macrophages from an anti-inflammatory “type 2” (M2) to a pro-inflammatory “type 1” (M1) state, the latter contributing to an increase in obesity [48]. Supernatants of M1 macrophages contain pro-inflammatory tumor necrosis factor (TNF) and IL-1beta, which inhibit the adipogenic differentiation of adipose tissue-derived stem cells [57]. Thus, M1 macrophages enhancing inflammation in adipose tissue is now considered to contribute to adipocyte dysfunction and may limit healthy fat growth [48].

**Figure 1:**
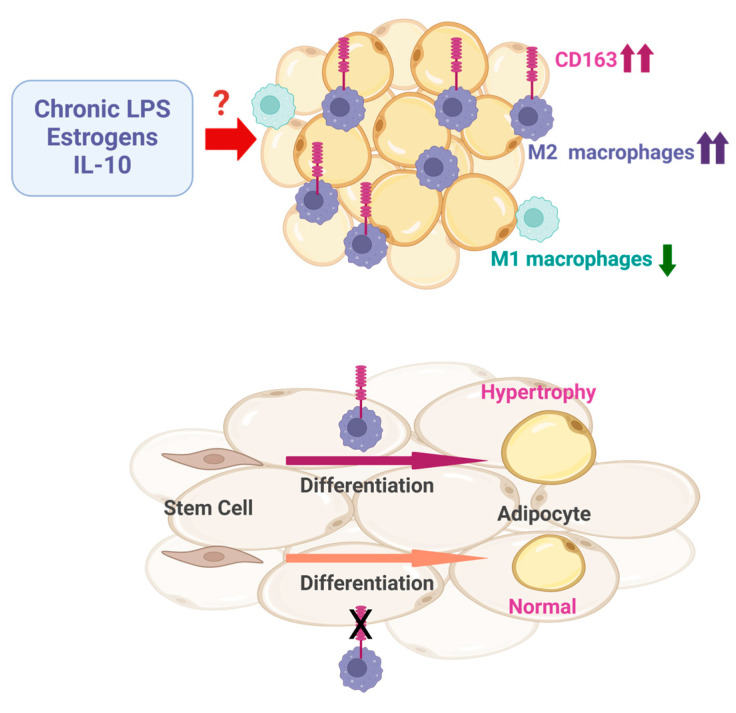
Lipedema adipose tissue is enriched with anti-inflammatory type M2 macrophages characterized by high CD163 expression levels. As the expression of markers for M1 macrophages is decreased, this indicates their reduced presence in lipedema tissue. The increased number of CD163-positive macrophages may be the result of chronic exposure to low levels of lipopolysaccharide (LPS), IL-10, or estrogens. M2 macrophages expressing the scavenger receptor CD163 contribute to increased lipid accumulation of adipocytes and cell hypertrophy. Created in BioRender. (2025) https://BioRender.com/h38r990, accessed on 19 February 2025.

Despite the link between inflammation and the pathophysiology of adipose tissue in obese settings, the expression of genes associated with inflammation was not increased in lipedema-derived stem cells or differentiated adipocytes [37]. However, a higher number of stromal vascular cells in liposuction material from patients with lipedema was reported, implicating an increased infiltration of macrophages in this tissue [40]. Indeed, adipose tissue from lean and obese lipedema patients, most of them in stage 2, was enriched in macrophages compared to controls [47,49]. Likewise, a higher expression of macrophage markers was also found in the subcutaneous fat of the thighs of stage 2 and 3 lipedema patients. Interestingly, these cells showed anti-inflammatory M2 macrophage polarization [15,47]. Obese adipose tissue is often characterized by crown-like structures, representing macrophages surrounding apoptotic adipocytes, and these were also present in lipedema [40]. To further investigate the composition of macrophages in adipose tissue from lipedema, anatomically comparable tissue samples from individuals with lipedema and controls with similar BMI were analyzed [47]. The SVF of stage 2 and 3 patients was enriched with macrophages expressing the scavenger receptor *CD163* [47]. This member of the scavenger receptor cysteine-rich superfamily is induced by anti-inflammatory cytokines (e.g., IL-6, IL-10), estrogens, and glucocorticoids yet downregulated by inflammatory stimuli, including TNF, interferon-γ, and lipopolysaccharides, and it commonly serves as a marker for M2 macrophages [58,59,60,61] (Figure 1). These findings suggested increased amounts of M2 macrophages in subcutaneous fat in lipedema. This hypothesis was further supported by RNA sequencing of macrophage marker CD11b-positive cells derived from adipose tissue of lipedema patients, identifying not only increased levels of *CD163* but also elevated expression of other M2 macrophage markers such as *CD301* and *PPAR-γ coactivator 1-beta*. Simultaneously, in these lipedema-related fat tissue samples, a decreased expression of marker genes for M1 macrophages, including *IL-6* and *IL-1 receptor 1*, was determined [47] (Figure 1). The significantly higher expression of CD163 in subcutaneous fat from lipedema patients compared to BMI-matched controls in other studies further points to increased amounts of M2 macrophages in subcutaneous lipedema fat [54].

CD163 is a transmembrane receptor mainly expressed on the cell surface of monocytes and macrophages, functioning as a scavenger receptor to remove hemoglobin and hemoglobin/haptoglobin complexes that form upon intravascular hemolysis, the degradation of which leads to the production of anti-inflammatory metabolites. As outlined above, CD163 expression is increased by anti-inflammatory mediators and cytokines, in particular IL-6, whereas proinflammatory cytokines decrease CD163 levels. [59]. Hence, anti-inflammatory cells expressing elevated levels of CD163 critically regulate the immune response [58,62].

CD163 expression on macrophages was also associated with increased lipid storage of adipocytes [47]. Adipose tissue-derived stem cells showed increased lipid accumulation compared to controls when differentiated in SVF-derived conditioned media of lipedema patients characterized by high expression levels of CD163. The treatment of the SVF from lipedema patients with IPI-549, a selective phosphatidylinositol-3 kinase γ inhibitor that switches M2 macrophages towards an M1-like phenotype, caused an almost complete downregulation of macrophage CD163. Moreover, these IPI-549-treated SVF cells promoted adipose-derived stem cell differentiation comparable to cells isolated from healthy controls. Further supporting CD163-expressing M2 macrophages as the driver of lipid accumulation in lipedema, treatment of the control SVF with IPI-549 had no effect on adipose-derived stem cell differentiation [47].

In monocytes, incubation with adiponectin led to a decrease in both cellular and surface CD163 levels, while the concentration of sCD163 in the corresponding supernatants remained unchanged [63]. The underlying mechanism is not fully understood, but adenosine monophosphate-kinase (AMPK) could be involved. Adiponectin activates AMPK [23], and this route may be relevant for lowering CD163 levels in adipose tissue, as metformin and 5-aminoimidazole-4-carboxamide-1-4-ribofuranoside (AICAR), both pharmacological modulators of AMPK, also downregulate CD163 expression [23,64]. Hence, alterations in adiponectin secretion from adipocytes in healthy or inflammatory/obese fat tissue may influence CD136 expression in infiltrating macrophages. However, as adiponectin levels are either comparable or elevated compared to controls (see Table 1), the relevance of these observations in relation to elevated CD163 levels in M2 macrophages of subcutaneous fat from lipedema patients has yet to be clarified.

Besides acting as a cell surface receptor, toll-like receptor ligands promote the shedding of the ectodomain of CD163, resulting in the release of soluble CD163 (sCD163), which further stimulates the cellular uptake of free hemoglobin [58,60]. Soluble CD163 is present in the bloodstream, and elevated sCD163 levels are found in the serum of critically ill patients as well as in chronic inflammatory and infectious conditions [58,65], which raised the possibility that sCD163 levels could serve as a tool to support the diagnosis of lipedema. However, sCD163 levels in patients with lipedema were normal [47], suggesting that although high CD163 levels are commonly observed in adipose tissue and shedding of sCD163 is induced in inflammation, this may not occur in the adipose tissue of lipedema patients [37].

Low-grade inflammation in adipose tissue has been suggested to contribute to lipedema. Bacterial lipopolysaccharides as well as saturated fatty acids such as palmitate can activate the toll-like receptor 4 pathway, initiating an inflammatory signaling cascade [2]. Lipopolysaccharides induce CD163 shedding, and after an initial decrease in cell surface CD163, a marked increase was observed 24 h later [66]. Thus, constant exposure of cells to low levels of endotoxins may cause high levels of CD163 in lipedema (Figure 1). Saturated fatty acids such as palmitate may also contribute to inflammation, in part via the activation of toll-like receptor 4 [67,68]. However, the lipid composition of the lipoaspirates from seven controls and nine lipedema patients collected after 8 h of starvation was comparable [16], suggesting that saturated lipids released from adipose tissue under these conditions do not contribute to local inflammation and enhanced expression and the shedding of CD163 in lipedema patients. However, it remains to be determined if a chronic, low-grade inflammation exists in lipedema adipose tissues, possibly contributing to disease progression.

In addition to macrophages, other immune cells such as T lymphocytes are activated in obese adipose tissue [69]. Felmerer and coworkers analyzed the T cell compartment, in particular the CD4 + T cells, and showed that their numbers were comparable in lipedemic and control adipose tissue [31]. Similarly, comparable T cell and mast cell numbers in the adipose tissue of obese and non-obese patients and controls have been reported by others [49]. However, a more detailed characterization of the different immune cells in lipedemic tissue is still lacking and may help to clarify the role of the immune system in the pathology of lipedema.

## 5. Endothelial Cells and Complement

### 5.1. Endothelial Cells

Morphological changes associated with lipedema include increased permeability and fragility of the blood vessels, leading to an accumulation of fluid and protein in the affected adipose tissues. There is also the dysfunction of the lymphatic system, which can lead to secondary lymphedema, a recognized comorbidity of lipedema [2]. Indeed, endothelial cell dysfunction leading to increased capillary permeability and lymphatic fluid accumulation in tissues is a common morphological feature in patients with lipedema [2,3,5]. This is in line with an earlier study correlating lipedema with functional changes in the lymphatic system, as lipedema patients displayed an atypical lymphoscintigraphic pattern characterized by a slowing of lymphatic flow [70]. Others also observed an increase in lymphatic vessel and angiogenic areas in lipedema [49]. On the other hand, in a study with women with lipedema stage 2 or 3, the morphology of the lymphatic vessels was normal, excluding lymphatic dysfunction in the development of lipedema [31].

It remains uncertain whether the predominant factor for the dysfunction of the lymphatic system in lipedema is the increasing size of the adipocytes or an inherent problem within the interstitial space or microlymphatic pathway. However, in obesity, which is characterized by similar or even greater fat accumulation, a marked decrease in lymphatic flow is lacking [3] and the number of endothelial cells is not altered in the fat of obese people [71]. Adipose tissue expansion in obesity depends on vascular remodeling, including angiogenesis, which is essential for the increased adipocyte mass to obtain sufficient amounts of nutrients and oxygen [72]. In line with adiponectin stimulating adipose tissue proliferation and adipogenesis, lymphatic endothelial cells also respond to adiponectin, promoting lymphangiogenesis [73]. Adipocytes in the obese state express angiogenic factors such as *vascular endothelial growth factor* (VEGF)-C, which coincides with the elevated expression of the corresponding receptors in endothelial cells [71]. VEGF-A is essential for angiogenesis, and inappropriate activity hinders white fat expansion in obesity, leading to metabolic disease [74]. Macrophages infiltrating fat tissues actively stimulate angiogenesis, which subsequently enables adipogenesis, a process essential for adipose tissue growth [75].

Based on these insights on adipose tissue expansion in healthy and obese settings, these angiogenic factors and their receptors were determined in lipedema, identifying the decreased expression of *VEGF-A* and EGF-D, while *VEGF receptor-3* levels were increased, altogether suggesting macrophage infiltration [31] that may influence angiogenesis and subsequent adipose tissue expansion and function. Also, higher serum levels of VEGF-C in patients with lipedema [31] were observed, yet these findings need confirmation in larger cohorts. Interestingly, mice with VEGF-D-induced lymphangiogenesis had higher fat mass and lower lean mass and were cold intolerant [76], and downregulation of VEGF-D may be considered a protective approach.

**Figure 2:**
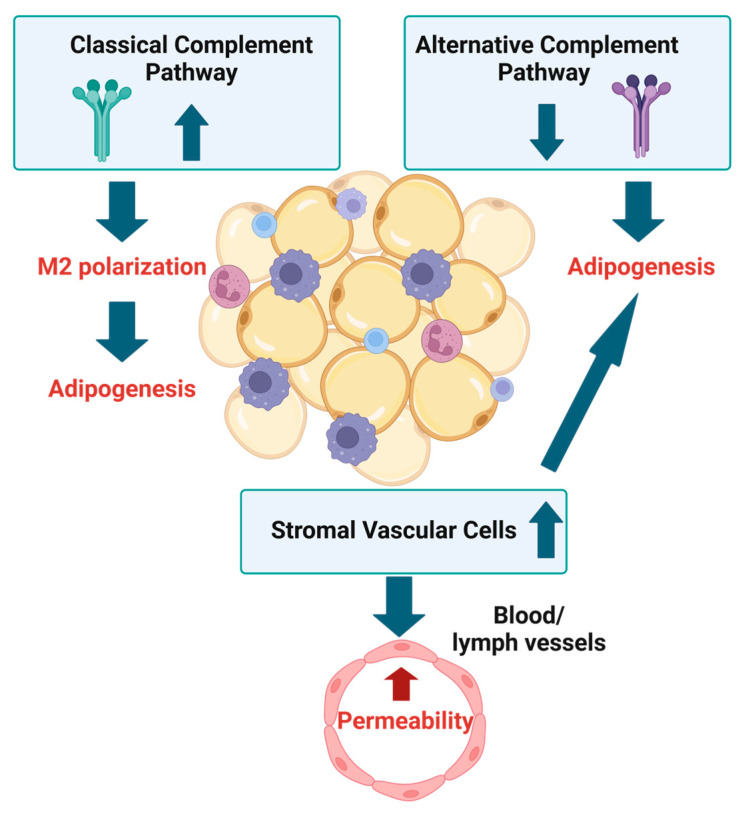
Lipedema adipose tissue has elevated stromal vascular cells, which can increase the permeability of vessels. In addition, stromal vascular cells were also shown to promote adipogenesis. Higher levels of the components of the classical complement pathway can induce M2 macrophage polarization, shown to enhance adipogenesis. Lower levels of the components of the alternative complement pathway may increase adipogenesis. Created in BioRender. (2025) https://BioRender.com/n48i771, accessed on 19 February 2025.

Prospero homeobox 1 (Prox1) is required for the development of the lymphatic system [77,78]. The deletion of Prox1 in lymphatic endothelial cells caused moderate defects in the lymphatic function, and these mice became obese with age [77,78]. This study indicates that lymphatic dysfunction may contribute to the accumulation of adipose tissue in patients with lipedema. Other underlying causes compromising endothelial cell function in lipedema may include yet-to-be-identified factors secreted from the SVF, as the secretome of the SVF from lipedema patients causes endothelial cell dysfunction, which may increase capillary permeability, suggesting that the stromal vascular cells initiate disease pathology [51] (Figure 2).

Along these lines, the increased expression of CD31, a vascular endothelial marker [37], and the endothelial/pericyte marker CD146 in SVF isolated from lipedema patients compared to healthy controls have been reported [40,49]. Pericytes, together with vascular smooth muscle cells, are classified as mural cells. These cells surround the endothelium of both blood and lymphatic vessels and provide essential support for their function [79]. It has yet to be determined if the higher number of these cells in the SVF of patients with lipedema contribute to disease pathophysiology.

Ketone bodies metabolized by lymphatic endothelial cells belong to the list of intermediate biomolecules that regulate the expression of lymphatic genes such as *Prox1*, *VEGF receptor-3*, and *VEGF-C* [80,81]. Ketones increase the levels of metabolites such as acetyl-CoA in lymphatic endothelial cells and increase vascular sprouting and angiogenesis. Notably, a high-fat, low-carbohydrate ketogenic diet increased lymphangiogenesis in mouse models and decreased edema [81]. Ketogenic diets improved clinical features in patients with lipedema [19] and may be developed as a nutritional therapy. Further research should unravel if this diet has similar effects on the lymphatic systems as those reported in mice [81].

Exosomes are small extracellular vesicles containing macromolecules such as lipids, proteins, microRNAs, growth factors, and cytokines that are released by cells to serve as mediators for cell–cell communication in both physiological and pathological settings. Hence, exosomes and their cargo have been proposed as candidates for the identification of biomarkers for the early diagnosis of a variety of human pathologies [82]. A mass spectrometry analysis identified elevated levels of platelet factor 4 in exosomes isolated from the plasma of patients diagnosed with lipedema (n = 15; stage 1 (n = 4), stage 2 (n = 6), stage 3 (n = 5); BMI 36 ± 9 kg/m^2^) compared to the controls [83]. Platelet factor 4 levels were also elevated in patients with lymphedema and those with developmental disorders of the lymphatic system [83]. Taken together, these findings suggest an association between lipedema and lymphatic abnormalities [83], and exosomal platelet factor 4 may become a biomarker for the diagnosis of this disease [83]. Notably, platelet factor 4 induces monocytes to differentiate into macrophages with the irreversible loss of CD163 [62]. Given the high CD163 levels on macrophages of subcutaneous fat tissues from lipedema patients [47,54], this suggests that this potential exosomal biomarker for lipedema does not contribute to the macrophage population found in the adipose tissue of these patients.

### 5.2. Complement

The complement system emerged early in evolutionary history and comprises a plethora of proteins (n > 50) that contribute to the innate immune response [84]. The classical complement pathway is initiated by the binding of the complement component 1q (C1q) to pathogens or antigen–antibody complexes. The alternative complement pathway targets pathogens or their by-products such as LPS [2,84]. A crucial element mostly generated by the alternative complement pathway is the C5b-9 protein complex, which facilitates the formation of transmembrane channels that lead to cell lysis [2,84]. The C5b-9 complex also evokes M1 polarization of macrophages [85].

Both the classical and alternative complement pathways converge on the proteolytic cleavage of C3, the most abundant complement protein in the circulation, by C3 convertases. The activation of C3 results in the production of the opsonin C3b, which labels target surfaces, including those of apoptotic cells. The smaller fragment produced upon C3 cleavage, C3a, has several immunomodulatory roles in both immune and non-immune settings [84]. Targets opsonized with C3b modulate the polarization of macrophages into M2 cells, whereas C3a induces proinflammatory cytokines and induces M1 polarization [85].

Adipocytes produce components of the classical and alternative complement pathways that induce inflammatory responses to counteract infections [2,86]. A transcriptomic analysis of subcutaneous fat from patients and BMI-matched controls showed a significantly higher expression of complement *C1q A, B*, and *C* chains in stage 2 lipedema [54]. A decrease in the expression of *complement factor D* (adipsin), which is involved in the alternative complement pathway, was observed in the fat of patients [15].

Low activation of the alternative complement pathway can initiate adipogenesis, and high activity can lead to adipocyte pyroptosis, a form of programmed cell death associated with cell rupture, causing the release of cellular contents that can activate strong inflammatory and immune responses [2] (Figure 2). Dead cells opsonized with C1q favor the polarization of macrophages to an M2-like phenotype [85] (Figure 2). The impact of the complement system on adipogenesis and M2 polarization of macrophages in lipedema warrants further study.

### 5.3. Emerging Therapies for Lipedema

Current therapeutic approaches for lipedema emphasize symptom relief and are based on thorough decongestive therapy. [5]. The adipose tissue of patients with lipedema usually does not respond well to exercise and conventional diets [87], and up to date, specific dietary approaches have only been studied in rather small patient cohorts. A study comparing a medium-fat/medium-carbohydrate diet with a high-fat/low-carbohydrate diet reported better body fat loss and reduction in limb circumference with the latter intervention [88]. A ketogenic diet also improved clinical features in patients with lipedema [19] and may have the potential to be developed as a nutritional therapy. The benefits of a modified Mediterranean diet consisting of seasonal fruits and vegetables, whole grains, legumes, and nuts, replacing saturated fats with unsaturated fatty acids and salt with herbs and spices, for 4 weeks were also reported [89]. Patients lost adipose tissue of legs and arms and experienced reduced pain, fatigue, and anxiety [89]. Dietary supplements with antioxidant effects such as hesperidin and diosmin (flavanone glycosides from citrus fruits) have also been suggested for the treatment of lipedema [90]. However, the effectiveness of different diets or supplements has not been validated in larger cohorts, and the small size of most lipedema trials remains a limitation.

Semaglutide, approved in 2018, is a synthetic analogue of glucagon-like peptide-1 and plays a critical role in regulating glucose metabolism and gastrointestinal motility. Studies have shown that in the obese, semaglutide leads to a substantial weight loss and reduction in waist circumference compared with a placebo [91]. However, this medication has not been tested in lipedema.

The blockage of the CD163 receptor on the surface of adipose tissue macrophages by suitable antibodies may represent a reasonable approach for lipedema therapy. However, to test the in vivo application of this concept, a lipedema mouse model to better understand disease pathology and test for therapeutic strategies is still lacking [87]. Hence, the clinical use of CD163 inhibitory antibodies remains to be developed. Along these lines, a challenge for this approach is the need to not interfere with CD163 expression in peripheral monocytes and macrophages localized in non-adipose tissues essential for the uptake of haptoglobin–hemoglobin complexes [58].

## 6. Conclusions

Lipedema studies are limited, and an inadequate number of participants further complicate therapeutically meaningful interpretations. It is well known that sample size is critical in clinical research, and experimental outcomes and conclusions, in terms of p-values, are less reliable when based on small cohorts [46]. Thus, larger multicenter trials are recommended for the future. Lipedema is a common condition affecting mainly women during hormonal changes and represents a complex, yet poorly understood dysfunction involving macrophages, adipocytes, and endothelial cells. Impaired lymphatic endothelial function and increased permeability of lymph and blood vessels are early signs of dysfunction and disease onset. The identification of diagnostic markers for lipedema remains a challenge, but platelet factor 4 in plasma-derived exosomes is elevated in lipedema and has high sensitivity and specificity to discriminate patients with lipedema from controls. Other characteristic features in fat tissue of lipedema patients include the increased adipogenic potential of adipose tissue-derived stem cells, the higher number of stromal vascular cells, and the increased amount of M2 macrophages. These macrophages express the scavenger receptor CD163, a marker protein of M2 cells, the blockade of which normalizes the adipogenic potential of adipose-derived stem cells. Components of the complement system, which are abundantly expressed in adipose tissue of lipedema patients, may contribute to M2 polarization. Treatment options remain to be established, but a ketogenic diet, which may act by improving lymphatic endothelial cell function, as shown in mice, improves clinical symptoms and pain in patients with lipedema. Further research is required to dissect the cell types and mechanisms involved in the development of lipedema.

## Figures and Tables

**Table 1:** Comparison of several adipokine levels in serum/plasma of patients with lipedema and controls.

Stage	Cases	Controls	BMI (kg/m^2^)	Adipokine levels (Case vs. Control)	Reference
Lymphedema-lipolymphedema Mild (n = 2) Moderate (n = 7) Widespread (n = 9) Severe (n = 1)	19	15	Case 34.4 ± 7.7 Control 25.5 ± 2.7	Comparable: adiponectin, visfatin, resistin	[30]
Stage 3 (n = 9) Stage 2 (n = 2) Stage 1 (n = 2)	13	13	Case 32.6 ± 5.8 Control 32.5 ± 6.0	Higher: adiponectin Comparable: leptin	[14]
Stage 3 (n = 7) Stage 2 (n = 2) Stage 1 (n = 1)	10	11	Case 27.2 ± 2.2 Controls 28.4 ± 3.9	Comparable: IL-6, lipocalin-2, leptin	[15]

**Table 2:** Adipocyte size of patients with lipedema and controls.

	Cases	Controls	BMI (kg/m^2^)	Adipocyte Hypertrophy	Reference
Stage 3 (n = 5) Stage 2 (n = 7) Stage 1 (n = 6)	18	12	Cases 27.0 ± 4.1 Control 26.6 ± 4.2	Yes: only stages 2 and 3	[38]
Stage 3 (n = 7) Stage 2 (n = 2) Stage 1 (n = 1)	10	11	Cases 27.2 ± 2.2 Controls 28.4 ± 3.9	Yes	[15]
Stage 3 (n = 1) Stage 2 (n = 7) Stage 1 (n = 8)	16	10	Cases 25.2 ± 0.5 Controls 23.5 ± 0.7	Yes for lean lipedema patients	[49]
Stage 3 (n = 2) Stage 2 (n = 12) Stage 1 (n = 1)	15	9	Cases 34.1 ± 1.4 Controls 34.1 ± 0.8	No	[49]
Stage 1–2 (n = 5)	5	4	Cases 29.5 ± 2.5 Controls 23.3 ± 3.0	No	[51].
Stage 3 (n = 9) Stage 2 (n = 10) Stage 1 (n = 1)	20	10	Cases: 27.6 ± 2.5 Controls: 27.9 ± 4.2	Yes	[16]
Stage 3 (n = 4) Stage 2 (n = 6)	10	10	Cases: 29.1 ± 3.8 Controls: 27.9 ± 4.5	Yes	[52]

## Data Availability

Not applicable.

## Abbreviations

The following abbreviations are used in this manuscript:

BMI	Body mass index
CRP	C-reactive protein
IL	Interleukin
PPAR-γ	Peroxisome proliferator-activated receptor gamma
Prox1	Prospero homeobox 1
TNF	Tumor necrosis factor
VEGF	Vascular endothelial growth factor
